# The Prevalence and Etiology of Surgical Site Infections Following Gastrointestinal Tract Surgery: A Cross-Sectional Study From a Tertiary Care Hospital

**DOI:** 10.7759/cureus.27320

**Published:** 2022-07-26

**Authors:** Kazim Raza Khan, Jaya Kumari, Syed Muhammad Waqar Haider, Shaikh Basiq Ul Fawwad, Narindar Kumar, Rukhsar Nizar, Deepak Kumar, Sangam ., Mohammad Hasan, Hassan Mumtaz

**Affiliations:** 1 Department of General Surgery, Jinnah Postgraduate Medical Centre, Karachi, PAK; 2 Internal Medicine, Mehran Medical Center, Karachi, PAK; 3 Department of Internal Medicine, Jinnah Sindh Medical University, Karachi, PAK; 4 Department of Internal Medicine, Liaquat National Hospital and Medical College, Karachi, PAK; 5 Department of Internal Medicine, Dow University of Health Sciences, Civil Hospital Karachi, Karachi, PAK; 6 Department of Internal Medicine, Jinnah Postgraduate Medical Centre, Karachi, PAK; 7 Urology, Guy’s and St Thomas’ NHS Foundation Trust, London, GBR; 8 General Practice, Surrey Docks Health Centre, London, GBR; 9 Public Health, Health Services Academy, Islamabad, PAK; 10 Clinical Research Center, Maroof International Hospital, Islamabad, PAK

**Keywords:** gastrointestinal surgery, infection control, surgery general, general infectious diseases, surgical site infections

## Abstract

Background

Surgical site infection (SSI) is the most commonly occurring infection in postoperative patients. This study is conducted to evaluate the prevalence of SSI in patients following gastrointestinal tract surgery and identify the risk factors.

Method

A cross-sectional study was conducted at the Jinnah Postgraduate Medical Centre (JPMC), Karachi, Pakistan, between December 2021 and May 2022. A total of 132 patients participated in the study who were at least 18 years older and had undergone the gastrointestinal surgical procedure. Patients who refused to give consent, died after the procedure, and were diagnosed with SSI after they were discharged were excluded from the study. We performed a chi-squared test.

Result

A total of 132 patients were included in the study, of which 63 (47.7%) were males, while 69 (52.3%) were females. SSI was more commonly found among the age group of 41-70 years with 29 (38.7%) patients. Presurgical features including hemoglobin of >11 mg/dL, albumin of >3.5 g/dL, blood glucose of <80 mg/dL, and emergency surgery were found to be associated with the SSI having a significant p-value. Similarly, the surgical and postsurgical features significantly associated with the SSI having a significant p-value were the presence of surgical trauma, wound irrigation with normal saline, malignancy, bowel preparation, longer duration of the surgery, intraoperative hypotension, operative site, drain insertion, and the absence of a second antibiotic.

Conclusion

The early identification and management of the demographical, presurgical, surgical, and postsurgical risk factors can help reduce the incidence of SSIs. Bowel preparation should be encouraged, and unnecessary delays during the surgical process leading to increased procedure time should be avoided. Extra precaution needs to be provided for the patients highly susceptible to SSIs.

## Introduction

Surgical site infection (SSI) is defined by the Centers for Disease Control and Prevention (CDC) as wound infection after an operative procedure at the site of an incision, space, or organ [1]. The European Centre for Disease Prevention and Control (ECDC) also defines SSI as an infection that occurs within 30 days after a surgical procedure without an implant [2]. The report of the World Health Organization (WHO) suggests that SSIs are the second most commonly hospital-acquired infections mostly in the USA and Europe, increasing the length of stay in the hospitals and leading to an extra financial burden on the healthcare systems [3]. The incidence of SSI was found to be higher in developing countries as compared to developed countries. Surveillance of SSI after discharge was done in 2019 on patients who had undergone clean and clean-contaminated operative procedures, and the incidence was found to be 15% in lower-middle-income countries [4].

SSI is reported to be one of the most commonly occurring infections following all surgical procedures with a prevalence of 1%-3%[5]. The increasing prevalence of surgical site infections contributes to a burden on the healthcare system all around the world, specifically the healthcare system of lower-middle-class countries [6]. Multiple studies suggest that the incidence of SSIs is quite higher in abdominal surgeries, with a prevalence of around 15%-25% depending on the extent of contamination, as compared to other types of surgeries [5,7-9].

The etiological factors for the development of SSI are quite variable. The major factors according to the Centers for Disease Control and Prevention and National Nosocomial Infections Surveillance System SSI risk index are the American Society of Anesthesiologists (ASA) score, wound classification, and longer specific surgery duration [10]. A study reported that the use of single-incision laparoscopic surgery (SILS) is safe and technically feasible for patients who were undergoing colorectal surgery [11]. The occurrence of SSIs is mainly dependent on the surgical procedure, environment of the healthcare facility, and level of care. The patient suffering from SSI is associated with delayed wound healing, pain, and discomfort that leads to longer stays in the hospital and can also cause death [12]. Based on the perspective of the surgeons, the associated etiology and the treatment outcomes of patients who had undergone a surgical procedure might have a direct relation to the occurrence of SSIs [13].

This study aims to evaluate the prevalence and associated risk factors leading to SSI in patients undergoing gastrointestinal tract surgeries in our population and determine the effects it has on the length of postoperative days. There is a knowledge gap in addition to a lack of studies in Pakistan related to the incidence of SSIs. Therefore, this can help surgeons identify the prevalence of SSIs and the modifiable risk factors that can be eliminated to lower the incidence of the SSIs, which can ultimately lower the burden on the healthcare system.

## Materials and methods

A hospital-based cross-sectional study was conducted at the general surgery departments of Jinnah Postgraduate Medical Centre (JPMC), Karachi, Pakistan, from December 2021 to May 2022. JPMC is one of the largest tertiary care public hospitals in the region. Ethical approval was sought from the institutional review board (IRB) committee of Jinnah Postgraduate Medical Centre (reference number: No.F.2-81/2022-GENL/21150/JPMC).

The sample size for this study was calculated using the Raosoft sample size calculator where the margin of error was 5%, the confidence level was 95%, and the expected population of the cases was 200 [14]. The sample size was calculated to be 132. The non-probability convenient sampling technique was employed to recruit the patients in this study.

The targeted population consisted of all patients with ages ranging from 18 to 70 years of both genders who were admitted to the general surgical wards to undergo gastrointestinal tract surgery, both elective and emergency cases. Patients who expired during the hospital stay and those who refused to give consent were excluded from our study. Patients who developed SSI after being discharged from the ward and then were readmitted for treatment were also excluded from the study. Every patient eligible to be included was followed from the time of admission to the time of discharge using a structured questionnaire to collect relevant data. Verbal informed consent was taken from the patients.

Data were collected using the established study protocol. The variables related to the patient’s demographic characteristics and preoperative features included age, sex, American Society of Anesthesiologists (ASA) score, body mass index (BMI), presence of any comorbidities (diabetes, hypertension, chronic liver dysfunction, chronic cardiac dysfunction, and immunosuppressive medications), smoking status, previous surgical history, urgency of surgery (elective or emergency), surgical approach (open or laparoscopic), class of wound, and values of hemoglobin, albumin, and blood glucose measured six hours before starting the operation. All variables including surgical and postoperative features are mentioned in the tables. Informed verbal consent was taken from all patients.

The patients were followed in the ward until their discharge. The assessment of wounds was conducted based on the definition of SSI devised by the Centers for Disease Control and Prevention and the National Healthcare Safety Network [1]. The surgical site infection was categorized as superficial (only skin and subcutaneous tissues involved), deep (involvement of deeper soft tissues such as muscle layers and fascia), or organ space (involvement of any anatomical part that was opened or manipulated during the surgical procedure).

Data were collected and then entered in SPSS Statistics version 26 (IBM Corp., Armonk, NY, USA) to be appropriately analyzed. The frequency of the categorical variables was calculated and mentioned in the respective tables. The association between the independent categorical variables and the incidence of SSIs was measured using the chi-squared test. A p-value of <0.05 was considered significant.

## Results

A total of 132 patients were enrolled in our study from the time of their admission to the date of their discharge, among which the demographic of the patients included 63 (47.7%) male patients and 69 (52.3%) female patients. Twenty-nine (38.7%) patients in the age group of 41-70 years were diagnosed with surgical site infection (SSI). In patients with SSI, nine (64.3%) patients had no formal education, 23 (36.5%) patients had a BMI within the range of 25-29.99 kg/m^2^, 31 (43.7%) patients had previous surgical history, and 23 (37.7%) patients were former smokers. The age, educational level, BMI, previous surgical history, and smoking history were related to the SSI with a significant p-value (Table 1).

**Table 1:** Association of demographical features with SSI SSI: surgical site infection

Variables	Categories	Number of patients		p-value
		With SSI (n (%))	Without SSI (n (%))	
Age	18-40	11 (19.3%)	46 (80.7%)	0.016
	41-70	29 (38.7%)	46 (61.3%)	
Gender	Male	17 (27%)	46 (73%)	0.428
	Female	23 (33.3%)	46 (66.7%)	
Education	No formal education	9 (64.3%)	5 (35.7%)	0.001
	Matriculation	9 (50%)	9 (50%)	
	Higher secondary	3 (14.3%)	18 (85.7%)	
	Graduation	8 (16.3%)	41 (83.7%)	
	Postgraduation	11 (36.7%)	19 (63.3%)	
Residential area	Rural	22 (37.9%)	36 (62.1%)	0.091
	Urban	18 (24.3%)	56 (75.7%)	
BMI	18.5-24.99	13 (35.1%)	24 (64.9%)	0.042
	25-29.99	23 (36.5%)	40 (63.5%)	
	>30	4 (12.5%)	28 (87.5%)	
Comorbid	Diabetes	9 (24.3%)	28 (75.7%)	0.315
	Hypertension	6 (37.5%)	10 (62.5%)	
	Chronic liver disease	5 (31.3%)	11 (68.8%)	
	Chronic kidney disease	3 (23.1%)	10 (76.9%)	
	Chronic heart disease	9 (40.9%)	13 (59.1%)	
	Tuberculosis	1 (7.7%)	12 (92.3%)	
	Immunosuppressive medications	4 (40%)	6 (60%)	
	No comorbidity	3 (60%)	2 (40%)	
Smoking history	None	10 (58.8%)	7 (41.2%)	0.00
	Former	23 (37.7%)	38 (62.3%)	
	Current	7 (13%)	47 (87%)	
Previous surgical history	Yes	31 (43.7%)	40 (56.3%)	0.00
	No	9 (14.8%)	52 (85.2%)	

The number of patients who reported having SSI was 40 (30.3%). The most common type of SSI was deep SSI with 18 (45%) patients (Table 2).

**Table 2:** Incidence of SSI

Variables	Categories	N (%)
Incidence of SSI	Yes	40 (30.3%)
	No	92 (69.7%)
Type of SSI	Superficial	11 (27.5%)
	Deep	18 (45%)
	Organ space	11 (27.5%)
Timing of SSI relative to the operation	Day 1- 3	4 (10%)
	Day 4- 6	22 (55%)
	One week or more	14 (35%)
Culture and sensitivity	Yes (+)	35 (87.5%)
	Yes (-)	2 (5%)
	Not performed	3 (7.5%)

In the group of patients with SSI, 26 (42.6%) patients had hemoglobin more than 11 mg/dL, 26 (38.2%) had albumin more than 3.5 g/dL, 12 (42.9%) patients had blood glucose less than 80 mg/dL, and 27 (38%) patients had undergone emergency surgery. The hemoglobin, albumin, blood glucose, and type of urgency were the preoperative variables that were found to be related to the SSI with a significant p-value (Table 3).

**Table 3:** Association of preoperative features with SSI

Variables	Categories	Patients		p-value
		With SSI (n (%))	Without SSI (n (%))	
Hemoglobin	Less than 11 mg/dL	14 (19.7%)	57 (80.3%)	0.004
	More than 11 mg/dL	26 (42.6%)	35 (57.4%)	
Albumin (g/dL)	Less than 3.5	14 (21.9%)	50 (78.1%)	0.041
	More than 3.5	26 (38.2%)	42 (61.8%)	
Blood glucose (mg/dL)	<80	12 (42.9%)	16 (57.1%)	0.011
	80-200	22 (36.7%)	38 (63.3%)	
	>200	6 (13.6%)	38 (86.4%)	
Type of urgency	Elective	13 (21.3%)	48 (78.7%)	0.037
	Emergency	27 (38%)	44 (62%)	
Approach	Laparoscopy	14 (23.3%)	46 (76.7%)	0.112
	Open	26 (36.1%)	46 (63.9%)	
Class of wound	Clean	10 (35.7%)	18 (64.3%)	0.478
	Clean-contaminated	10 (40%)	15 (60%)	
	Contaminated	6 (27.3%)	16 (72.7%)	
	Dirty	14 (24.6%)	43 (75.4%)	
ASA score	1	8 (50%)	8 (50%)	0.002
	2	15 (34.1%)	29 (65.9%)	
	3	15 (39.5%)	23 (60.5%)	
	4	2 (5.9%)	32 (94.1%)	

In the SSI group of patients, 31 (43.7%) patients had a surgical trauma, 12 (36.4%) patients had wound irrigation with saline, 28 (45.2%) patients were operated on for a malignant condition, 29 (46.8%) patients had a bowel wall preparation, 33 (52.4%) patients had a duration of surgery for more than three hours, 10 (14.7%) patients developed intraoperative hypotension, 11 (50%) patients had an operational site on the large bowel, 28 (41.4%) had a drain insertion, and 40 (36.4%) were not given a second antibiotic. The presence of surgical trauma, wound irrigation with a saline, malignant surgical condition, large bowel as an operational site, drain insertion, bowel preparation, longer duration of surgery, a second antibiotic given, and intraoperative hypotension were significantly associated with the occurrence of SSI having a significant p-value (Table 4).

**Table 4:** Association of surgical and postsurgical features with SSI

Variables	Categories	Patients		p-value
		With SSI (n (%))	Without SSI (n (%))	
Surgical trauma	Yes	31 (43.7%)	40 (56.3%)	0.00
	No	9 (14.8%)	52 (85.2%)	
Wound irrigation	Saline	12 (36.4%)	21 (63.6%)	0.008
	Povidone solution	3 (13%)	20 (87%)	
	Hydrogen peroxide	17 (26.6%)	47 (73.4%)	
	None	8 (66.7%)	4 (33.3%)	
Suture material	Silk	15 (39.5%)	23 (60.5%)	0.055
	Absorbable	21 (32.8%)	43 (67.2%)	
	Antimicrobial	4 (13.3%)	26 (86.7%)	
Surgical condition	Malignant	28 (45.2%)	34 (54.8%)	0.00
	Nonmalignant	12 (17.1%)	58 (82.9%)	
Bowel preparation	Yes	29 (46.8%)	33 (53.2%)	0.00
	No	11 (15.7%)	59 (84.3%)	
Duration of surgery	Less than three hours	7 (10.1%)	62 (89.9%)	0.00
	More than three hours	33 (52.4%)	30 (47.6%)	
Intraoperative hypotension	Yes	10 (14.7%)	58 (85.3%)	0.00
	No	30 (46.9%)	34 (53.1%)	
Operation site	Small bowel	9 (34.6%)	17 (65.4%)	0.00
	Large bowel	11 (50%)	11 (50%)	
	Pancreatic	1 (4.3%)	22 (95.7%)	
	Biliary	19 (31.1%)	42 (68.9%)	
Drain insertion	Yes	28 (41.8%)	39 (58.2%)	0.00
	No	12 (18.5%)	53 (81.5%)	
Incisional protection	Gauze	9 (30%)	21 (70%)	0.791
	Adhesive drapes	4 (21.1%)	15 (78.9%)	
	Wedge edge protectors	20 (31.7%)	43 (68.3%)	
	None	7 (35%)	13 (65%)	
Intraoperative blood loss	No blood loss	10 (34.5%)	19 (65.5%)	0.821
	Less than 500 mL	17 (30.4%)	39 (69.6%)	
	More than 500 mL	13 (27.7%)	34 (72.3%)	
Duration of postoperative antibiotic	One day	8 (53.3%)	7 (46.7%)	0.082
	2-4 days	10 (38.5%)	16 (61.5%)	
	5-7 days	4 (18.2%)	18 (81.8%)	
	>7 days	18 (26.1%)	51 (73.9%)	
Second antibiotic given	Yes	0 (0%)	22 (100%)	0.001
	No	40 (36.4%)	70 (63.6%)	
Hospital stay	Less than seven days	13 (22%)	46 (78%)	0.063
	More than seven days	27 (37%)	46 (63%)	

## Discussion

This study aimed to document the prevalence of SSI in the tertiary care hospital in Pakistan and evaluate the modifiable and non-modifiable risk factors. These factors are vast in number, and the commonly discussed factors include malnutrition, obesity, the use of steroids or any other immunosuppressive medication, poor skin preparation, bowel preparation, longer duration of surgery time, hypothermia, age, diabetes, hypoalbuminemia (<3.5 g/dL), and the inappropriate use of antibiotic prophylaxis [15]. In our study, the identifiable risk factors were divided into demographic, preoperative, surgical, and postoperative factors, among which the modifiable risk factors included in our study were preoperative hemoglobin, glucose, and albumin levels, smoking status, BMI, educational status, surgical approach, wound irrigation, suture material, bowel preparation, intraoperative hypotension, duration of the surgery, drain insertion, incisional protection, postoperative antibiotic duration, and length of hospital stay. A significant association was found between higher BMI, no formal education, smoking history, longer duration of the surgery (more than three hours), presence of surgical trauma, intraoperative hypotension, bowel preparation, and smoking status with the incidence of SSI in our setup. Therefore, any unnecessary delays need to be avoided during the surgical procedure, precautions should be taken to avoid surgical trauma, and intraoperative hypotension needs to be managed appropriately. To decrease the duration of the surgery, the surgeon’s education must be optimized, and an experienced paramedic staff should be appointed. Bowel preparation should be developed as a standard protocol. The prevalence of SSIs can be reduced if risk factors are appropriately identified and the required preventive measures are taken [16]. In one study, the artificial neural network analysis to predict SSIs revealed the complexity of the risk factors and stated that specific factors such as wound classification are more significant as compared to other risk factors [17]. However, the wound classification was not significantly related to SSI in our study.

The male gender was reported to be the most affected gender in other studies [18,19]. However, gender was not significantly associated with SSI in our setup. In our study, 30.3% of the patients who underwent gastrointestinal surgery were diagnosed with SSI. The prevalence is quite consistent with the findings of the study conducted by Hassan et al., where the prevalence of SSI was found to be 27.1% [19]. In our study, the major variables that were found to be significant risk factors leading to the development of the SSI were previous surgical history, the presence of surgical trauma, malignant surgical condition, and longer surgical duration. The longer hours of the surgery and the malignant nature of the disease were also the significant risk factors in the study by Hassan et al. [19]. The age group affected most by SSIs was the elder group of patients aged 41-70 years, which is comparable with the study by Ling et al. [20]. SSIs develop most commonly within a week in our study, which is consistent with the findings of the study conducted in Sudan [19].

Of the patients with malignant diseases developing SSIs, 45.2% is justifiable as the malignancy weakens the immune system and makes the patient vulnerable to infections. This finding is consistent with several other studies as well, where 30%-60% of patients develop SSIs after surgeries for colorectal cancer [21-23]. Hence, in the group of patients on immunosuppressive medications, 40% developed SSIs in our study.

The major limitation of this study was its unicentric approach, due to which limited data was collected. A multicentric approach for this study would have included more variables into consideration, and broader data would have been acquired. The small sample size was another significant limitation that would have been avoided if the duration of the study would have been extended or if the data was collected from other hospitals. Another important limitation of the study was excluding the data of the readmitted patients. The inclusion of more variables such as grading of the surgeons and the involved nursing staff in the operation theater should also be taken into account to identify how it affects the incidence of SSIs in future studies.

## Conclusions

This study concludes that the early identification and management of the demographical, presurgical, surgical, and postsurgical risk factors can help reduce the incidence of SSIs within the hospital setup. Bowel preparation should be encouraged, and unnecessary delays during the surgical process leading to increased procedure time should be avoided. Extra precaution needs to be provided for the patients highly susceptible to SSIs.
